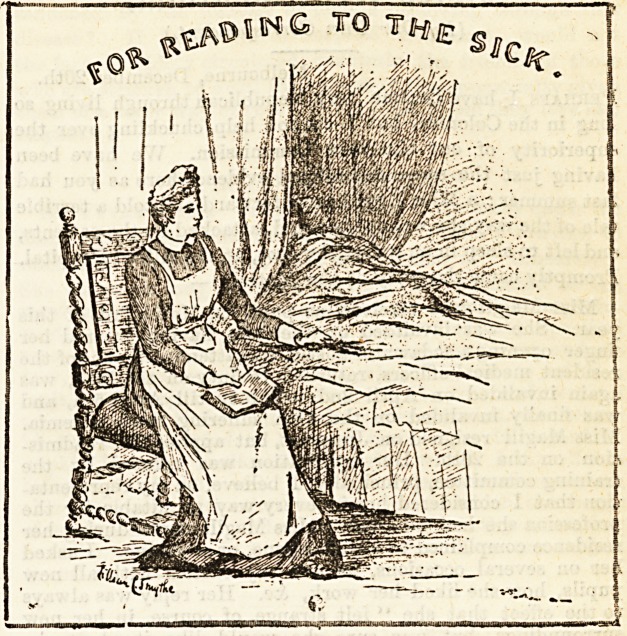# Extra Supplement—The Nursing Mirror

**Published:** 1891-02-14

**Authors:** 


### "The Hospital" Nursing Mirror.

Being the Extra Nursing Supplement of "The Hospital" Newspaper.

Contributions for this Supplement should be addressed to the Editor, The Hospital, 140, Strand, London, W.C., and should have the word "Nursing" plainly written in left-hand top corner of the envelope.

### En Passant.

THE NURSES' CO-OPERATION.—Any doubts about the success of the Nurses Co-operation have been promptly dispelled by its first ten days' work. As we expected the medical profession has recognised the movement as one worthy of support, and the demand for nurses has been greater than the supply. In fact, more nurses are badly needed at the Co-operation. The pretty little bedrooms are now ready for those nurses who prefer to live at the office, and the Lady Superintendent is at home daily from ten to four. Anyone interested in the movement is invited to call; the address is 8, Cavendish Street, Portland Place, W.

KEEPING CHRISTMAS.—We thought we had done with this heading, but it is not to be, for we must make mention of the treat to the little patients of the Evelina Hospital, which was held on January 29th. The wards were brightly decorated, and the usual tree yielded presents to all. At Lyme Regis the pretty Cottage Hospital, which is built on a hill overlooking the sea, the patients had extra fare and friends to see them, and every effort was made to make the season as happy as possible. At the Coldstream Cottage Hospital there was a pleasant entertainment, magic lantern and songs, following a substantial meal. Captain Baillie Hamilton, the Rev. P. Mearns, Miss Henderson, and other visitors were present.

SHORT ITEMS.—A new paper, *Pearson's Weekly*, reproduced in its 29th number an article entitled "A Nurse's Day in Hospital," which appeared in the *St. James's Gazette* in April, 1888. The editor of one of these papers has good cause for complaint.—The attendants of Banstead Asylum have subscribed towards a fund for the relief of local distress.—There is an article by Sister Rose Gertrude in the *Ladies' Home Journal* for February.—Some pretty nurses in uniform handed the programmes at the dance at the Portman Rooms on the 3rd inst., in aid of the North-West London Hospital.—The B. N. A. has applied to the Board of Trade for permission to become an incorporated institution.—The Johannesberg Nurses' Home has been closed under painful circumstances.—Probationers have great difficulty in getting accepted at Bartholomew's just now, because of the very severe medical examination their health is subjected to by the staff, owing to the late illness amongst the nurses.

SUNDERLAND NURSING INSTITUTE.—The Mayor presided at the annual meeting of this Institute, which was well attended, especially by ladies. The report was a good one. The demand for nurses had been so great that the Institute had only been able to supply half the number of applications, and the Committee were anxious to increase the number of nurses as rapidly as might be found possible. At the beginning of the year the staff consisted of nine nurses and one matron. During the year eight nurses—of whom four were of their own training—joined, and five resigned, leaving twelve nurses on the staff. There were at present nine probationers being trained, and it was hoped to add considerably to their number during the year. The tables setting forth the work done in the twelve months showed that in the department of district nursing there had been 82 patients, the number of visits paid being 1,809. In the private nursing branch there had been 92 cases, and the amount of the earnings ot the nurses was £477 5s. 6d.

BELFAST NURSES.—The District Nursing Society of Belfast is entirely managed by a committee of ladies, and the current report shows the touch of feminine fingers. It is no mere formal statement; it branches off to details of the work and the cases, and has a few remarks on General Booth, the first appearance of influenza in Ulster in 1326, &c. The society has done good work for 18 years, and its financial position is satisfactory. Let us hope the next 18 years will be as productive of good work as those that are passed.

BIRMINGHAM DISTRICT NURSES.—Birmingham is so busy about its new hospital just now, that the district nurses are likely to be overlooked, though they are in particular need of help. The influenza spared them no more than others; indeed, it made extra patients, and reduced the number of available nurses—a most illogical proceeding. With sisterly generosity the Birmingham and Midland Counties Training Institution came forward with a grant of £56, which enabled two extra nurses to be put on. The accounts for the year showed £292 0s. 6d. received from subscriptions, £21 0s. 6d. from donations, and £85 5s. 7d. from the hospital collection, and there was a deficit of £55 18s. 4½d. on the year's working.

TRURO NURSES' HOME.—This institution seems to be somewhat hampered for want of funds and capital; during the past year as many as sixteen applications for nurses have been refused in one month. It is found difficult to get good nurses to go to Cornwall, say the Committee; we can quite believe it, unless they get good wages, and as the Institution only charges a guinea a week for their services, and a guinea and a half for infectious cases, the wages are small. Two guineas ought to be the least charge for infectious cases. The Home proposes to train its own probationers, a very good plan, and we would advise them at once to secure more capital and carry it out. The Lady Superintendent is overworked, and the nurses have not too much leisure; the Home would surely do well then to raise its charges, make its staff more comfortable, and forsake for the present the plan of starting a district nurse. Is it true that the Truro nurses are expected to do a good deal of laundry work?

THE LIFE OF A NURSE.—Last week the *Daily Graphic* had two delightful and accurate articles describing the daily routine of nursing life. They had sent their "Lady Commissioner" to St. Thomas's, Guy's, and Middlesex, and they had also sent their lady artist, and the result was a true picture of the pleasures as well as the trials of a nurse's life. From probationer to staff-nurse, thence to sister, the Lady Commissioner followed the career of a nurse, and then quaintly remarked that so far "our nurse has not died of starvation"! Indeed, we could quote with pleasure a large part of these bright and sensible articles, only we fancy that most of our readers must have seen them. Therefore, we conclude with just these two sentences:—"The best nurses resent continual outside clamour concerning themselves and their hours, saying that they knew what they were undertaking before they embarked upon the life, and never expected it to be all play. In fact, they regard it rather in the light of an insult to their own intelligence and powers of concerted action, for they are perfectly well aware that they could redress any genuine hardship for themselves."

### Lectures on Surgical Ward Work and Nursing.

By Alexander Miles, M.B. (Edin.), C.M., F.R.C.S.E.

Lecture XIII.—OPERATIONS *(continued)*.

The Lotions.—(1) Prepare a basin of carbolic (1 20) for purifying the skin of the patient, and for the surgeon's hands. In it should be placed a nail brush, and a swab of wool. (2) Two basins (enamelled) of corrosive lotion (1-2000) for irrigating during operation, a swab of wool in each. These must be changed so soon as ever a brownish deposit appears in the lotion. (3) Two or more basins of carbolic for washing sponges, also to be frequently replenished. (*Vide* under "Sponges.") All the lotions should be diluted with tepid water. In preparing these make a rule always to put the *lotion* into the basin first, so that should the other ingredient by any chance be forgotten, your error shall be on the side of antiseptic safety.

The Basins.—I have already indicated the necessity of using enamelled basins for corrosive lotion, and also the importance of purifying the edges of kidney and bleeding tins, before bringing them in contact with a wound.

The Carbolised Towels.—A number of these should be prepared with 1 in 20 lotion, and wrung out just as they are required. You cannot make them too dry. They should be handed up spread out on a macintosh, and should cover over the whole area of operation. Always have a few spare towels and macintoshes to prepare if required. Towels sterilised by heat have certain advantages over carbolised towels, but their use has only been attended with partial success.

The Sponges.—Remove the sponges from the jar of 1 in 20 carbolic in which they are kept, and after squeezing them dry, place them in a solution tin, covered over with a dipped towel. During the operation they must be handed up to the surgeon as dry as they can possibly be made. When received back from the operator they are saturated with blood, and it is now your duty to cleanse them of this. Now if you plunge them at once into carbolic lotion, the albumen of the blood will be coagulated, and the small stringy masses of fibrin produced are only removed with great difficulty. To obviate this, you should first wash out all the blood in plain water, and subsequently pass the sponge through 1 in 40 and then 1 in 20 carbolic, before allowing it to be used again. Be particularly careful never to hand up a sponge direct from the plain water, as that is not antiseptic. I have already warned you that corrosive sublimate blackens sponges. Always count your sponges before operations in which a large cavity is opened, for example, the abdominal or thoraxic cavities, and mark the number in use on a slip of paper. On no account tear a sponge during the operation, and before the wound is closed, count them again lest any should be left inside. If a sponge fall on the floor, or into the sawdust box, you must put it aside altogether, and must not permit it to be used again till it has been thoroughly purified. In cases in which the discharge is putrid, sponges should be discarded and swabs of wool used instead.

Cleansing of Sponges after Operation.—The sponges should be taken from the lotion as soon after the operation as possible, and all the blood and pus removed from them by repeated washing with hot water, until the water remains unstained by blood. They are then transferred to a strong solution of carbonate of soda (washing soda) and left there for twenty-four hours that all grease may be dissolved out. At the end of that time they are again washed in clean water and replaced in 1 in 20 carbolic, till again required. It is a good plan to keep two complete sets of sponges so that a periodical purification can be carried on without interruption. The following directions for cleansing sponges which have been in use for some time, are given by Caird and Cathcart in their excellent "Surgical Handbook":—(1) Free them from grease by steeping in a concentrated solution of washing soda; (2) Then soak for twenty-four hours in permanganate of potash 1 gr. to 1 oz., and wash again in clean water; (3) Soak in 1 per cent solution of commercial salt of sub-sulphite of soda, with 8 per cent pure concentrated hydrochloric acid (in 24 oz. of water, ℨi of the soda, and ℥ii of the acid) until (in about a quarter of an hour) they have become white; (4) Again wash in water until scentless, and store in 5 per cent. (1-20) carbolic acid.

Preparation of Operation Sponges.—It may be convenient here to give some directions as to how sponges are made fit for use in surgical operations. Having secured Turkey sponges of the best quality, very close, and about the size of a closed fist, these should be thoroughly washed in hot water and then dried. They must next have all the sand and calcareous particles removed from them. This is done by pounding them with a mallet on a firm table to reduce the grits to a powder, and then washing this out by alternately passing them through very hot and cold water till the latter remains quite clean. The beating should be carried on for half an hour at a time, and must be repeated till not a single gritty particle is to be found in the sponge. They are then placed in 1 in 20 carbolic where they should remain for at least a fortnight before being used, the lotion being changed once or twice in that time.

To prepare sponges for sponge-grafting, the finest sponge should be chosen. The calcareous particles are removed by steeping it in dilute nitrohydrochloric acid, and any excess of acid is removed by means of a dilute solution of potash or ammonia. The sponge is then antisepticised by 1 in 20 carbolic, and is ready for use.

The nailbrush, turpentine, and soda, are used for purifying the skin of the part to be operated upon. The carbolised towel, which is always applied for some hours before operation, softens the superficial epitheluin, and by means of powdered soda and the nail brush, this effete epitheluin is dissolved and removed, and with it the manifold germs contained in it. Follow this with spirit of turpentine, which is a good solvent of fat and oil in addition to being an antiseptic.

The Drainage Tubes.—The most commonly used material is india-rubber tubing perforated along its sides at intervals of about three quarters of an inch, the diameter of the holes being about a third of the circumference of the tube. Decalcified bone, and strands of cat-gut have the advantage of being absorbable, and therefore not necessitating dressing of the wound for their removal. Glass and metal tubes have also been used All drainage tubes should be kept in glass jars, about six inches high and four inches in diameter, with close-fitting stoppers, and should be immersed in 1 in 20 carbolic. Always ensure that a drainage tube cannot slip into a wound and be retained there. This is very simply done by transfixing it with a sterilised safety pin, a stock of which should be kept in carbolic for the purpose.

### Appointments.

Cambridge Home for Nurses.—Miss Young has been appointed Lady Superintendent. She was trained at King's College Hospital, and had charge of wards there and at Charing Cross for some years. For the last nine years she has been Sister of the male medical ward at Addenbrooke's Hospital, Cambridge, where her valuable services as medical sister and instructress of the probationers have been much appreciated. Her resignation of this post has caused universal regret.

C. C. Hospital, Irvine.—Miss M. A. Fraser has been appointed Head Nurse of the Cunningham Combination Hospital. She trained at Aberdeen Royal Infirmary for four years, and for the last five years has been working at the Barnhill Hospital.

Gibraltar.—Miss Caroline Walker and Miss Henrietta Whiteford, both trained at the London Hospital, have sailed for Gibraltar, having been appointed sisters of the Government Civil Hospital there.

Melbourne.—Miss M. E. Stephenson, known and respected at the Launceston Hospital as Sister Edith, has gone to Australia, having been appointed Matron of a hospital at Melbourne.

### The Midwives Bill.

The nature and extent of the opposition to the Midwives' Bill is shown in the published returns to the circular (asking for their opinions on the Bill) sent out in October last by the Chairman of the Parliamentary Bills Committee of the British Medical Association.

A considerable number of branches are stated to have replied; the names of seventeen are given. Of these one (Yorkshire) is opposed to both principle and Bill. Three leave the matter in the hands of the Committee; three more are satisfied with the Bill as it is. Two objected to the Bill in its present form, seven considered that the clauses required amendment, one (the Reading and Upper Thames branch) also appears to have objected to some of the present clauses.

It is thus practically universally agreed that midwives should be registered, and their practice limited and defined by law.

The objections to the Bill may be classed under four heads:—(1) Education and examination; (2) definitions; (3) details; (4) prosecutions.

1. One point raised is as to the amount of knowledge a woman practising as a midwife should possess, who, being uncertified, desires to be placed on the register. This is admittedly a difficult question; encouraging examination without requiring it seems to be a liberal and just way of settling it. The midwife's patients will be better off through her being under the law, and the midwife will feel that she must acquire some amount of knowledge. Some, at least of the most ignorant will have taken up midwifery through inability from ill-health to perform laborious work. The Bill requires a certificate of good health. There will, at least, be every inducement for them to acquire knowledge. Another point raised is as to foreign diplomas. There are very few foreigners practising as midwives in England, and those mostly among their own countrywomen, and it seems a little absurd to exclude foreigners from the register if the standard of their diploma is equal to that of one obtained in England, and if practising before the formation of the register, they could not be excluded from it. Another difficulty lay in the choice of the body to whom the duty of examination should be confided. The County Councils hardly seem to be the right bodies, and probably the suggestion of the Council of the Obstetrical Society will prove more acceptable. It is that a strong• central examining board should be formed in Loudon, with ten or twelve local examining boards in connection with it.

2. Definitions of the terms "natural labour" and "midwife" are asked to be included in the Bill. The one naturally goes with the other, and definition of a natural labour has been kept back by the Association's Committee from the Privy Council, it having been found that, although it could be defined, this was not easily done.

3. As to the details, most of them have already been agreed to by the Committee, which is now reconsidering the clauses of the Bill.

4. It is also said that prosecution of a midwife would be impossible under the present Bill, as permission has to be obtained from one of certain specified bodies before this could be begun. If this had been done in the case of the Medical Act what an amount of uncalled for annoyance, expense, and litigation would have been prevented Profiting by the past is the best that can be done. It is to be remarked that of the eighteen objections stated to have been raised to various clauses of the Bill, twelve of them come from the Birmingham and Midland Counties Branch, and the Stafford-shire Branch, and three more from the Reading and Upper Thames Branch, leaving three only for the remaining branches of the Association.

It appears, therefore, that the opposition to the Bill, of which we have heard so much, has been limited to something like four branches of the British Medical Association, and a legal opinion on which the opponents of the Bill much relied (if one is to judge by the way in which it has been circulated) is now shown, as all persons with any common sense must have recognised, to be based on bad law, and to be built up with worse logic. This legal opinion stated that a midwife would be on a par with, and capable of being registered in the Medical Register, in defiance of the fact that three qualifications are required previous to being thus registered.

### BAD NIGHTS.

Sleep is one of the great blessings of life we never appreciate until we miss it; until some illness or trouble falls upon us, and that rest we had regarded as a certainty becomes fitful and uncertain. The night was meant for sleep and rest, just as the day was meant for work, but there comes a time to most of us when the natural order of things fails, and we are out of health, out of sympathy and touch with Nature. Then the fever runs high as the darkness descends, the pain becomes more unbearable, the power of stillness seems absolutely to depart.

All things assume a very terrible aspect by night: we look at the sufferers in the other beds in the ward and almost grudge them their rest, and we are bound to repress our groans or we would disturb and pain them.

What help, what comfort is to be had to make a bad night bearable?

This, that it can draw us nearer to Christ, and teach us more strongly the lesson of submission and patience. More or less the world is quiet during the hours of darkness, and it is easier to raise one's thoughts to the world beyond. Those who have wrestled with pain throughout the night have often learnt the name of an angel before the coming of the dawn. There have even been men who have rejoiced in their bodily suffering, because they have felt in it the hand of Christ. It is hard to toss feverishly whilst others rest; it is still harder to keep silence under our suffering so that we may not awake others; but oh! the "joy cometh in the morning," when the pain goes, and in the golden sunlight we seem to see the smile which rewards us for our fight. And though those hours of darkness and suffering are terribly long, there always comes the morning and the light at last, and there always comes the sense of fellowship with Christ, the Man of Sorrows. He knew long nights of pain and mental anguish, and if He has bidden us drink of a similar cup, shall we not solemnly, tremblingly, accept the pledge?

Those cannot really be "bad" nights which we spend with Christ; we must remember the beautiful lines:—

"He gives His angels charge of those who sleep,
But He, Himself, watches with those who wake."

### National Pension Fund for Nurses.

The Countess of Cadogan has been elected a Patroness of this Fund, to fill the vacancy caused by the lamented death of Lady Rosebery.

### Notes from Australia.

(*By Our Own Correspondent.*)

Melbourne, December 20th.

Perhaps I have become very Republican through living so long in the Colonies, but I cannot help chuckling over the superiority of our Charities Commission. We have been having just the same sensational evidence here as you had last summer; a Miss Magill came forward and told a terrible tale of the way she was overworked, attacked by the patients, and left to sleep in an unhealthy spot, at the Alfred Hospital. Promptly came the reply of the Matron :—

Miss Magill was received as pupil on March 3rd, of this year. She was invalided on the 8th, having injured her finger opening a sodawater bottle, was attended by one of the resident medical officers, returned to duty on the 15th, was again invalided on April 2nd, off duty till April 5th, and was finally invalided on the 14th suffering from anæmia. Miss Magill resigned on June 1st, but applied for readmission on the 26th. Her application was refused by the training committee, principally, I believe, on my representation that I considered her in every way unsuitable for the profession she had adopted. Miss Magill never during her residence complained to me of anyone, or anything. I asked her on several occasions, as was my custom with all new pupils, how she liked her work, &c. Her reply was always to the effect that she "felt strange, of course, in her new surroundings, but was sure she would like it when she became accustomed to it." Miss Magill never did scrubbing, and was never asked to do so, there being handmaids amongst whose duties it was included. I will not trespass upon your valuable space by taking each of Miss Magill's statements and replying to them; they are either untrue or so grossly exaggerated as to be unworthy of notice.

Well, our Commissioners are prompt and to the point; they arose in their might, and walked over to the Alfred Hospital, and some of them smiled gently as they carefully disproved some of the feminine facts of the would-be nurse who had turned out a failure. Now, why didn't your noble lords walk into the nearest hospital to lunch one day, and so disprove the stories of the bad food? But perhaps they are not enthusiastic enough to experiment on their own "vile bodies."

However, we have only one grumbler in our ranks, whereas you apparently mustered three in London. Miss Kate Williams, a nurse at the Alfred Hospital for five years, stated that Miss Magill's evidence was greatly exaggerated. Mrs. M. Caffyn, late of the Nightingale Home, St. Thomas's, London, and of the Metropolitan and National Nursing Association, spoke of the necessity of improving the status of nurses as much as possible. In Victoria it seemed as if every woman who could make a poultice or sponge a typhoid patient believed she was a competent nurse. The high standard of nursing recognised at home had hardly begun to be understood here. No thorough nursing system could be satisfactorily carried on without such a standard; otherwise nursing degenerated into a mixture of "Sairey Gampism," bad distribution, and general pauperism, and in the case of private nursing into a rather degraded mode of earning a livelihood and collecting gossip. Nursing in Victoria was twenty years behind the times. Dr. Caffyn gave evidence with regard to the abuse of the out-patient department, which is a far greater evil here than in England. Except among old immigrants, there is no justifiable poverty in this country. Miss Sutherland gave evidence on numerous points. She is a philanthropic worker, and on the question of neglected children spoke well, but her remarks on nursing and medical subjects betrayed woeful ignorance, and the Women's Hospital authorities utterly deny Miss Sutherland's facts. It is strange that people cannot be content to speak about things they know.

This subject is a fascinating one, but I must give a few items of general news in conclusion. We have a man trying a forty days' fast just now; he is a native of Sydney. The annual dinner of the Medical Society was held in November; Dr. Jackson was in the chair, and about thirty members were present. The plans for the enlargement of the Alfred Hospital have been approved; the new building will cost £7,000. A terrible tragedy has occurred at Ballarat; a man smothered four of his children and shot his wife, in an attack of mania. The lesson to be learnt from this is our need of private asylums. A wife will suffer almost anything rather than send her afflicted husband to herd with the riff-raff of maniacs in a public asylum; then these awful tragedies happen. Mrs. Strong and Miss Martelli are fighting a tremendous battle at St. Kilda, for the public, in its ignorance, objects to their private hospital. The question is now going before the Supreme Court, and doubtless these two valiant nurses will win, but we are all anxious that they should also obtain a heavy claim for costs.

### A Winter's Holiday.

### III.—THE THIRD WEEK.

At last an evening came when, after an elaborate study of guide-book and map, we planned an excursion which would take up the whole of our third week. It really was an ambitious effort, for we knew no Spanish, and we contemplated a trip into Spain. Our route was marked out, and our bags packed, and we went to bed with that pleasurable excitement which marks the anticipation that something nice was to befall us on the morrow.

Before noon next day—such a sunny day, too!—we were standing in front of a Bayonne inn, watching a leisurely man and a still more leisurely boy pack, unpack, and repack a most miscellaneous collection of baggage outside a rather decrepid-looking diligence. This being the "Courier," we had an idea that it might start punctually, but it did not fulfil this expectation, and we should have had time to enjoy with ease the scenes through which we had just passed so rapidly, the booths and the stalls by the river, with their gaily-coloured goods and the equally attractive costumes of the sellers.

At last the baggage was considered secure, heavy as some of it seemed to be. A huge tarpaulin was spread over all and the driver made his appearance on the scene. He was an enormously stout man, a handsome black-eyed Basque, who had travelled the same road for at least twenty years, and could boast that the only accident which had befallen him or his diligence in all that time took place during a very awful and unusual storm.

His appearance on the pavement was the signal for the arrival of his four horses, which were soon put to. Shabby-looking, lean beasts, but of considerable strength and speed.

Directly the last buckle was fastened we were invited to mount, which we did to the seat beside the coachman. Here we found comfortable space for the three of us. The Basque naturally required a good half of it for his comely proportions. A mile from the town, however, a fourth passenger appeared, who evidently objected to any place in the diligence save and except the one next to the driver, so he, too, squeezed himself in.

Happily, No. 4 was not only thin, but seemed a modest and quiet youth, so we all bore the close quarters with philosophy.

After a rather long silence the young Frenchman confided to us the fact of his possessing some knowledge of English, he having once passed six months very happily in a London suburb.

Our big driver proved a fund of entertainment, and he knew and greeted every man, woman, child, and dog whom we encountered. The beauty of the country through which we passed was above description—the winding river far below us, and mountains on all sides, whilst avenues of poplars, silver-barked birches, pollards, etc., bordered the road itself. The brilliant sunshine lasted all day, and we constantly congratulated ourselves at the foretaste of summer which we were enjoying in mid-winter, and when we reached the town of Cambo, we seemed to have found the embodiment of spring. My thoughts have often returned to that charming spot, and I never can realise the possibility of any change of seasons taking place there—it remains to me as a vision of perpetual spring-tide. It looked a place to "rest and be thankful" in, not a sphere for the work and the struggles of life.

After leaving Cambo, we saw more and more of the mountains, and our way lay up and down steep hills, which we traversed at such a pace that a kind of nervous admiration was developed in us for these gaunt horses to whom a stumble seemed impossible.

After passing through a few small towns and several villages without stopping, we came to Hasparen, where the horses were changed and we had some tea, an unusual luxury to find at a French country inn. This town did not seem specially interesting, but perhaps our stay was too limited for us to have time to discover its attractions.

We were soon again on our way, and in spite of our cramped positions and a good deal of jolting, the clear sweet air soon banished all weariness.

Somewhere between Hasparen and S. Jean Pied de Port, we came to the foot of a very steep hill where a boy suddenly appeared with a pair of oxen which he rapidly attached in front of our four horses and thus helped the diligence up this and one or two other equally severe ascents. He then vanished as suddenly as he had arrived.

These oxen are certainly beautiful creatures, handsome, strong, and gentle. Report says that they are so affectionate that if one of a pair dies, the other must be slaughtered immediately and sold for food, otherwise it would slowly pine away and die.

We reached S. Jean Pied de Port at ten, and bidding farewell to our civil travelling companions we passed through the now deserted streets to the lodging which had been recommended to us. The house proved to be a delightful old place with a charming outlook, although we naturally did not discover this advantage till we opened the wooden shutters early next morning.

The river flowed beneath the window, shallow and clear, crossed by a bridge just below us; opposite stood a church which was approached through a handsome archway, and further away lay the rest of the town.

### Everybody's Opinion.

[*Correspondence on all subjects is invited, but we cannot in any way be responsible for the opinions expressed by our correspondents. No communications can be entertained if the name and address of the correspondent is not given, or unless one side of the paper only be written on.*]

### THE DEATH-RATE IN TYPHOID.

MISS MADELINE FLETCHER writes: As a constant reader of your valuable paper, I should like to ask, Can any reason be assigned for the greater mortality in some infectious hospitals than others? Is it the diet, or nursing, or the different treatment by doctors, or ventilation? I have been particularly struck with the number of deaths from typhoid. Is it that I have been more fortunate than many? I was Matron and Head Nurse of the St. William's Infectious Hospital, Rochester, for four and a-half years, and also since I have been here as Matron and Head Nurse, and I have never yet lost a case of typhoid, and I have had under my charge some of the worst cases. And may I ask if it can be proved, and by what results, the numbers who recover from typhoid through private treatment at their own homes, and those who are treated at our infectious hospitals, as one is saddened by the number of deaths occurring through this disease? If in favour of hospital treatment, would not the fact, if widely circulated, constrain the friends of those who are stricken to avail themselves of the opportunity given them of a surer way of recovery for those whom they love and respect?

### INFECTIOUS HOSPITALS.

A "FEVER NURSE" writes: Being one of the many under the Metropolitan Asylums Board for some years, I venture to state that I cannot agree with the opinion of "Sincerity." She states as long as ward servants are promoted to the nursing staff, they are not qualified for nursing or superintending. Although not trained, I cannot see any specified reason why they are not, as a great many have been, and are now, educated, sensible women, who, if it can be called a fault, have begun at the bottom of the tree and worked upwards under their medical officer's supervision, and whose kindly aid has tried to forward their cause in regard to weekly lectures, medical and surgical work. I myself not being trained, but well versed in infectious diseases, am able to compete with other nursing, and in which the board of management has not deemed me an unworthy nurse, but one who tries to help bravely those whose lives are subject to serious diseases.

### Princess Christian's Daughter.

MISS E. DURHAM, Farringford, Freshwater, Isle of Wight, acknowledges the following subscriptions towards a wedding present for Princess Louise of Schleswig-Holstein, to be given as a proof of the gratitude of nurses for the interest Princess Christian has ever taken in their progress. Subscriptions will be received until the end of March, and will be acknowledged in these pages. Up to February 9th the following had been received:—

*Matrons* (each 5s.)—Miss Piggott, Miss Harris, S. H. C. *Sisters* (each 2s. 6d.)—C. H. Leslie, E. Garnett-Clarke, Rose Price, B.N.A., M.A. Barnes Groom, E. Bishop (3s.), Marie Rees, Head-nurse Noble. *Nurses* (each 1s.)—F.L. Elms, A Pro., L. M. Simmons, L. Stacy, P. Legate, A. E. Parkin, M. E. Higham, Isabella Thodey, Nurse Morris, A. S. Church, Nurse Frost, Nurse Dyke, Nurse B. E., Nurse V. H. M., M. Campbell, M. E. Waltham, E. Rorison, M. Buckton, S. Bagg, S. Watson, A. Clarke, L. Phillips, A. E. Coker, B.N.A., and E. Whitsey.

### Notes and Queries.

### Queries.

(34) *Fastening on Poultices.*—Is there any simple way of fastening on poultices to back and chest, other than bandaging?—*A Constant Reader.*

(35) *Nursing in New York.*—Is it difficult to get a post as Sister in a New York hospital, and can such a post be got before going out?—*Agatha.*

### Answers.

(29) *Air Cushions.*—In silk, can be had from John Weiss and Sons, 287, Oxford Street, price about 10s.

(30).—Apply to Nurse Child, Sussex County Hospital, Brighton.

(32).—Apply to Mrs. Armitage, 31, Raydon Street, Highgate, N.

*M. T.*—We pay no attention to anonymous contributions.

*N. A. R.*—Freshwater is in the Isle of Wight.

*E. B.*—Write to the Manager of THE HOSPITAL; he has published a Nurse's Case Book, price 1s.

*Sister May.*—The exhibition of dolls dressed in nursing uniforms is on view at this office (140, Strand), any day, except Saturdays, between ten and four.

*Sister H.*—You mark your letter "Private and Confidential," so that we cannot use it, and you use threats. If the communication you fear comes with name attached it will, of course, carry more weight than your letter. Probability is always on the side of those who do not fear publicity. We have always spoken well of the good work you have done, but your letter has damaged your cause far more than could the communication you wish us to suppress.

*Nurse H.*—There is no register yet. You can get all particulars from the Secretary, Midwives' Institute, 12, Buckingham Street, Strand, London.

*Trained Nurse.*—All applications in respect to Lady Roberts' Fund should be addressed Under-Secretary of State, India Office, Whitehall, London.

*Enquirer.*—Apply to Sister Katherine, St. Mary's Mission, Plaistow, E., or to Miss Derham, Caldicote House, Bushey, Herts.

### Nurse Hilary.

(*Continued from page* cvi.)

HILARY TYRWHITT's life of twenty-four years had seen much sunlight and also much darkness. The only child of a country doctor and his wife, she had passed a happy childhood, and a happy girlhood during which came to her, her life's crowning good, in her engagement to Alan Webster, a ward of the old vicar's—as fine, straightforward a young man as one could wish to see, and a general favourite in the village where he had been brought up. His strong, well-knit young figure, pleasant sunburnt face, and ready smile were always a welcome sight to all the country side. Open-handed, frank, and if somewhat careless—good-hearted to the core—it was no wonder; and life looked all that was bright for the present and securely happy for the future to the young couple and to the many loving and admiring folk around them. Alan was preparing for the Army; and it might have been through lack of concentration, certainly not from want of good intention that he failed in his first examination.

Then troubles, which truly never come singly, swept over them both. Alan's small fortune was lost to him through the dishonesty of one of his father's trustees. Dr. Tyrwhitt died very suddenly, and Hilary and her mother, now that the breadwinner was gone, found that they would have a hard struggle to live. Then Hilary bravely took up her arms and entered life's battle. Her girlhood's golden dream was over, and her womanhood's human reality had begun. She gave lessons, and in her spare time did needlework for sale, just managing to keep her mother and herself; and when her mother—always more or less of an invalid—died, about a year after her husband, Hilary, who inherited her father's taste for things medical, went into a London hospital.

Alan meanwhile, being unable to afford the necessary "crammers" had almost given up the idea of the Army, and kept the wolf from his door by copying and other work of the kind. Their love for each other was the red-gold streak that ran through these clouded sunset days of theirs, and gave them hope of a fair sunrise in the future, though, doubtless, a distant future they both knew.

"Well, good-bye, my dear," said Lady Beckett, after a half-hour's visit, "and to-morrow when you are off duty you must have a bit of a drive with me, and get some colour into your cheeks. To-day you have some other fish to fry, you say, so I won't say no more about it. I can't abide riding in the Victoria unless I've someone to keep me company, and your's is always welcome, love."

"Yes, thank you, I will," said Hilary, brightly, "I am sorry I cannot come to-day, but I must go somewhere else." There was a look of deep sadness on her face as she moved from bed to bed after Lady Beckett had gone, which the cheerfulness of her voice could not hide; and she closed her hand upon a certain letter which lay in her pocket as though the very touch of it would more fully realise all it meant to her.

It was from Alan: she knew it by heart now, and had repeated it to herself all the day long. It told her how, despairing of getting on in his present line of life, he had enlisted as a private; that his regiment was ordered out to the Soudan; and that he must leave London for Portsmouth at six o'clock this very evening.

"I must see you once more, sweetheart, before I go—there are a hundred things I want to say—and I cannot leave without one more look at your dear face. Sometimes I ask myself if it is right to keep you bound to such a struggling fellow as myself, and then I remember what you said once before when I told you that—and those words have been the life of my life and hopes ever since. Only come and say them again. I will meet you in the Green Park at four o'clock." There was much more in the letter—loving words which Hilary's heart chanted softly within itself, and which helped to brace it for the sad good-bye.

At half-past three she was "off duty," and a few minutes after she was coming down the stairs in her long cloak and simple little bonnet, when the doctor hastily appeared at the door of the Eleanor ward.

"You are the very person I want," he began, hurriedly. "A serious case has just come in—fall from the top of a 'bus. The head is severely injured, and she will want the most constant attention. Most likely an operation will be needed. I must ask you to come at once."

Hilary's heart stood still—her face whitened—she said rapidly, "Could not Nurse Alice take it? I shall not be long, and it is most important I should go."

"She *could*," he said, doubtfully; "but she has had so little experience in cases of the kind. It is unfortunate our being so short-handed just now. It will be touch and go in any case; only the most careful nursing can save her." He paused, and looked critically yet kindly at her.

"I will come," she said quietly; yet she clenched her hands and held her breath as though to brace herself desperately together as she turned and went up the stairs again.

(*To be continued.*)

### Where to Go.

THE St. John Ambulance Association holds lectures on "First Aid" and "Nursing" at 3, St. Bride Street, Ludgate Circus, commencing on February 27th. Further particulars can be had from Louis Jarman, Hon. Sec.—At St. Anne's Church, Soho, Bach's Passion Music will be given on Fridays during Lent. For tickets of admission send stamped addressed envelope to T. F. Curtis, 67, Frith Street, Soho.—At Marylebone Church the Passion Music will be given on Thursdays during Lent. For tickets send stamped addressed envelope to Mr. R. Mackworth, 64, High Street, W.

### Amusements and Relaxation.

### SPECIAL NOTICE TO CORRESPONDENTS.

First quarterly word competition commenced January 3rd, 1891; ends March 28th, 1891.

Competitors can enter for *all* quarterly competitions, but no competitor can take more than *one first prize* or *two* prizes of any kind during the year.

N.B.—Word dissections must be sent in WEEKLY not later than the first post on Thursday to the *Prize Editor*, 140, Strand, W.C., arranged alphabetically, with correct total affixed.

The word for dissection for this, the SEVENTH week of the quarter, being "VALENTINE."

| Names. | Feb. 5th. | Totals. | Names. | Feb. 5th. | Totals. |
|---|---|---|---|---|---|
| Reynard | — | 77 | Woodbine | — | 25 |
| Reldas | 39 | 232 | Madame B. | — | 25 |
| Tinie | — | 30 | Shakespeare | — | 59 |
| Patience | — | 76 | Smyrna | 24 | 129 |
| Jenny Wren | 24 | 204 | Southwood | — | 102 |
| Agamemnon | 38 | 223 | Gipsy Queen | — | 21 |
| Wyamaris | 38 | 223 | Snowball | — | 19 |
| E. C. | 39 | 228 | Rita | — | 88 |
| Ecila | 40 | 222 | Mortal | — | 16 |
| Hope | 37 | 226 | Nurse Annie | — | 15 |
| M. W. | 38 | 227 | Carmen | — | 11 |
| Qu'appelle | 38 | 225 | Grannie | — | 45 |
| Nil Desperandum | 40 | 223 | Amie | — | 30 |
| Lady Betty | 40 | 214 | M. R. | — | 25 |
| H. A. S. | 39 | 183 | Primrose | — | 24 |
| Sister Jack | — | 62 | Nurse J. S. | — | 38 |
| Crystal | — | 156 | | | |

## Figures and Tables

**Figure f1:**